# Dynamics and Conformations of a Full-Length CRESS-DNA Replicase

**DOI:** 10.3390/v15122393

**Published:** 2023-12-08

**Authors:** Elvira Tarasova, Reza Khayat

**Affiliations:** Department of Chemistry and Biochemistry, City College of New York, New York, NY 10031, USA

**Keywords:** all-atom Molecular Dynamics, CRESS-DNA, rolling circle replication, endonuclease, ATPase, helicase, ssDNA

## Abstract

Circular Rep-encoding single-stranded DNA (CRESS-DNA) viruses encode for a Replicase (Rep) that is essential for viral replication. Rep is a helicase with three domains: an endonuclease, an oligomeric, and an ATPase domain (ED, OD, and AD). Our recent cryo-EM structure of the porcine circovirus 2 (PCV2) Rep provided the first structure of a CRESS-DNA Rep. The structure visualized the ED to be highly mobile, Rep to form a homo-hexamer, bound ssDNA and nucleotides, and the AD to adopt a staircase arrangement around the ssDNA. We proposed a hand-over-hand mechanism by the ADs for ssDNA translocation. The hand-over-hand mechanism requires extensive movement of the AD. Here, we scrutinize this mechanism using all-atom Molecular Dynamics (MD) simulation of Rep in three states: (1) Rep bound to ssDNA and ADP, (2) Rep bound to ssDNA, and (3) Rep by itself. Each of the 700 nsec simulations converges within 200 nsec and provides important insight into the dynamics of Rep, the dynamics of Rep in the presence of these biomolecules, and the importance of ssDNA and ADP in driving the AD to adopt the staircase arrangement around the ssDNA. To the best of our knowledge, this is the first example of an all-atom MD simulation of a CRESS-DNA Rep. This study sets the basis of further MD studies aimed at obtaining a chemical understanding of how Rep uses nucleotide binding and hydrolysis to translocate ssDNA.

## 1. Introduction

More than half a century ago, Gilbert and Dressler coined the term rolling-circle replication (RCR) to describe the mechanism of genome replication by the bacteriophage ϕX174 [1]. Since then, RCR has been identified as a mechanism of replication by phage/viral genomes, bacterial plasmids, and eukaryotic Helitrons (transposons) [2,3,4,5,6,7,8,9,10,11]. The molecular mechanism of RCR is well documented; however, its structural mechanism is desperately lacking [12]. Eukaryotic Circular Rep-Encoding Single-Stranded DNA (CRESS DNA) viruses are a group of viruses that possess a circular single-stranded DNA (ssDNA) genome and utilize RCR for genome replication. CRESS-DNA viruses are diverse, widely prevalent, and infect organisms from the archaea, eubacteria, and eukarya domains of life [1,3,12,13,14]. The International Committee on Taxonomy of Viruses (ICTV) categorizes the *Cressdnaviricota* phylum into two classes, five orders, seven families, and thirty-two genera. The genome sizes for this phylum vary from 1 knt to 25 knt (https://talk.ictvonline.org/, accessed on 20 October 2023) [15,16]. CRESS-DNA viral genomes do not encode for a DNA polymerase. Consequently, replication of genome requires conversion of the ssDNA to double-stranded DNA (dsDNA) by the host’s machinery. It has been demonstrated that an oligonucleotide complementing the viral genome, and packaged into the viral capsid, acts as the primer for this conversion [17]. RCR of the dsDNA begins with a multifunctional viral-encoded initiator protein named replicase (Rep) that binds to a sequence specific origin of replication (*ori*). Bioinformatic, structural, and biochemical studies have demonstrated that Rep possesses three functional domains: an endonuclease domain (ED), an oligomerization domain (OD), and an ATPase domain (AD) [14,18]. ED is a member of the HUH (His-hydrophobic-His motif) endonuclease group, OD is homologous to homeodomain proteins, and AD belongs to the ATPase associated with various cellular activities (AAA+) super-family 3 (SF3). Binding to *ori* unwinds the double-stranded genome to generate a cruciform structure—a process that is possibly accompanied with ATP binding and hydrolysis by Rep [19]. The process of *ori* recognition and dsDNA reshaping remains to be described. The loop of the (+) strand cruciform is a nona-nucleotide sequence that is recognized by Rep’s ED. ED nicks this loop to generate a free 3′-OH end for leading strand DNA synthesis, and becomes covalently attached to the 5′-PO_4_ of the (+) ssDNA [12]. While the structural mechanism of ssDNA loop recognition has been described [20], the exact mechanism of nicking and Rep-ssDNA covalent modification remains to be described. Following strand nicking, Rep assembles as a homo-hexamer around the ssDNA [21]. It remains to be demonstrated whether the covalently modified Rep-ssDNA complex and the homo-hexamer encircling the ssDNA genome are the same. Rep then uses ATP binding, hydrolysis, and release to translocate ssDNA. The ssDNA translocation results in the unwinding of the dsDNA at the replication fork. The details of nucleotide binding, hydrolysis, and ssDNA translocation remain to be described. It has been postulated that Rep is responsible for recruiting the appropriate cellular components for DNA polymerization [19]. A little more than a full cycle of replication generates a complete (+) strand DNA with several nucleotides beyond the start site. The extended DNA is necessary for proper interaction with Rep. A second round of cleavage by an unmodified ED liberates the 3′-OH of the newly synthesized (+) strand. ED then acts as a ligase to ligate the 5′-PO_4_ (covalently attached to ED) to the recently liberated 3′-OH end to generate a circular ssDNA (+) strand. The (+) strand is packaged into the capsid. The (-) strand remains bound to a small oligonucleotide that serves as a primer for additional rounds of replication. It remains to be described if the same Rep complex is responsible for both nicking and ligation.

The *Circoviridae* family of viruses are members of the *Cressdnaviricota* phylum. These small nonenveloped icosahedral viruses possess circular ambisense ssDNA genomes. The *Circoviridae* family can be categorized into two genera according to capsid morphology and genome organization: the *Cyclovirus* and the *Circovirus*. Genetic material associated with the *Cyclovirus* have been identified from both vertebrates and invertebrates; however, a definitive host remains to be identified [22]. Members of the *Circovirus* are widely distributed in nature and have been documented to infect terrestrial, aquatic, and avian animals [23,24]. Porcine circovirus 2 (PCV2) is the prototypical representative of the *Circovirus* genus. Four types of PCV have been identified. These are morphologically similar but genetically and antigenically distinct. PCV1 is apathogenic and was first detected in porcine kidney (PK-15) cells [23]. PCV2 causes postweaning multisystemic wasting syndrome, later named porcine circovirus-associated disease or porcine circovirus disease, which culminates in the immunosuppression and eventual death of the host [24,25,26,27,28]. PCV3 has been associated with reproductive failure, nephropathy syndrome, and porcine dermatitis [29,30,31]. PCV4 was recently discovered in diseased pigs [32]. PCV2 has received the greatest attention as it is a detriment to swine industries, and outbreaks continue to occur in swine rearing countries despite a vaccination program [33,34,35]. A potential explanation for the continued outbreaks could be escape mutations accumulated by the capsid protein. Indeed, sequence analysis of the PCV2 capsid protein has identified eight genotypes (a–h) [36]. Primary factors driving the sequence diversity are likely to be recombination events and high mutation rates (1.2 × 10^−3^ substitutions site^−1^ year^−1^) of the PCV2 genome. This value is highest among DNA viruses and comparable to RNA viruses [37]. A candidate responsible for the reported mutation rates is the PCV2 Rep protein. RCR replication of the PCV2 genome requires Rep and a spliced variant Rep’ [38]. Biochemical studies have shown that Rep and Rep’ form hetero-oligomers involved in ssDNA processing; however, the interaction between Rep and Rep’, and the precise role of Rep’ in replication remains to be determined [39]. We recently determined the structure of the PCV2 Rep using cryo-electron microscopy (cryo-EM) with single particle analysis [18]. The structure demonstrated the following: (1) a highly mobile ED, (2) a hexameric oligomer, (3) an OD that is structurally homologous to homeodomain proteins, (4) six nucleotide binding sites defined by two adjacent ADs, (5) four of these sites to be occupied by ADPs and the remaining two sites empty, (6) the AD to be a member of the AAA+ SF3 [22], (7) a hexanucleotide ssDNA bound to the center of Rep and interacting with six AD through its phosphate backbone, (8) the AD arranged as a staircase around the ssDNA bound, and (9) a flexible C-terminus [18,21]. Our structure helped us propose that Rep followed the hand-over-hand mechanism described for AAA+ members that translocate substrate through their central pore [40]. In this mechanism, sequential ADs interact with the backbone phosphates of the ssDNA being translocated. Consequently, the subunits adopt a staircase arrangement to maintain their interaction with ssDNA. This arrangement results in a large gap between the subunit at the top and the bottom of the staircase. For the staircase arrangement (and substrate interaction) to be maintained, the subunit at the bottom of the staircase must release its hold from the ssDNA and translocate to the top of the staircase. Concurrently, the remaining AD and ssDNA translocate together the distance of one nucleotide down the staircase. This hand-over-hand motion requires nucleotide binding, hydrolysis, and release. The nucleotide binding sites are defined by adjacent AD. The hydrolytic state of the nucleotide (inferred from the binding interaction between the nucleotide and Rep) as one descends the staircase has been postulated to be ATP, ATP, ADP + Pi, ADP, empty, and empty [21].

We expand on our structural studies by asking what are the roles of ADP and ssDNA on the dynamics and conformation of PCV2 Rep? While cryo-EM is powerful in addressing slow (μsec-to-msec) conformational changes, faster conformational changes require an alternative approach. Consequently, we turned to using all-atom Molecular Dynamics (MD) simulation to address these questions. We performed a 700 nsec simulation of a full-length PCV2 Rep bound to (1) a 20 nucleotide (nt) ssDNA and ADP (ssDNA + ADP), (2) a 20 nt ssDNA, and (3) by itself. Our studies identify several important factors responsible for Rep’s activity. These include identifying (1) flexible local regions that are known to be important for Rep’s biochemical functions, (2) the ADP nucleotide that is ready for release in the next step of ATP binding–hydrolysis–release, (3) essential dynamics of Rep demonstrating global flexibility, (4) ssDNA and ADP altering the dynamics of Rep, and (5) ssDNA being responsible for establishing the staircase arrangement of AD.

## 2. Materials and Methods

### 2.1. Generating a Full Model of Rep Bound to ssDNA and ADP

Atomic coordinates for the six chains of Rep (GenBank: AAP44182.1) were generated by grafting the N- and C-termini of a Robetta server prediction onto experimentally determined structures [41]. The N-terminus of Rep was generated by superposing the Robetta model onto the crystal structure of ED (PDB entry 5XOR). Residues 14–110 of 5XOR (chain A) were grafted into the Robetta model because amino acids 14 and 110 from the two models exhibited overlap with rmsd < 1 Å. Subsequently, residues 49–56 of the Robetta model replaced the missing loop in 5XOR. Six of these models were overlaid onto the Rep cryo-EM (PDB entry 7LAR, residues 119–301) OD (residues 119–157) to generate an ED-OD hexamer. Residues 301–314 of AD were modeled by overlaying the Robetta AD onto each 7LAR AD then grafting amino acids 294–314 from the Robetta model into 7LAR. These amino acids were grafted because amino acids prior to this exhibited rmsd < 2 Å. A hexamer was generated by overlaying the ODs of ED-OD and OD-AD, then grafting each ED (residues 1–118) onto OD-AD. The OD hexamer of this model was overlayed onto the OD hexamer of the replication initiator protein of plasmid pMV158 (RepB) (PDB entry 3DKV). The RepB ED-OD is homologous to Rep [14]. The mainchain dihedrals of the PCV2 Rep residue 119 were manually adjusted using Coot to position the ED comparable to the ED in RepB and remove intermolecular clashing between the ED [42]. The “Regularize Zone” function of coot was used to automatically correct the geometry of regions where grafting occurred. The four ADP and Mg^2+^ from PDB entry 7LAR were included in the Rep-(ssDNA-ADP) simulation. The hexanucleotide ssDNA from PDB 7LAR was extended by 14 nt using the x3DNA server [43]. The four ADP and Mg^2+^ were removed from the Rep-(ssDNA-ADP) model to generate the Rep-ssDNA model. The ssDNA model was then removed to generate the Rep model.

### 2.2. Molecular Dynamics Simulation of Rep

The ACPYPE server was used to generate the ADP topology and parameter files [44]. The Amber99sb-ildn force field and the tip3p model for water were used for the simulation using the GROMACS 2020.3 package [45]. The net charge of the system was neutralized by the addition of 150 mM of Na^+^ and Cl^−^ ions. Steepest descent minimization (50K steps) was performed for each system with subsequent equilibration steps. Heavy atoms were restrained for equilibration: 20 ps of NVT ensembles followed by 20 ps of NPT ensemble. The final equilibration was performed by lifting all non-ssDNA atom restraints for 20 ns in NPT ensembles with 2 fs time steps. Positional restraints on the first and final atoms of ssDNA were maintained during the production run where temperature and pressure were kept at 300 K and 1 atm, respectively. Cutoff interactions were 12 Å for Van der Waals interactions and long-range Coulomb interactions (handled using the particle mesh Ewald algorithm) [46,47].

### 2.3. Trajectory Analyses

The periodic boundary condition correction was applied using the *trjconv* command with the center, ur, and compact options of GROMACS. Subsequent analyses were performed on the last 200 nsec of these trajectories or their extracted frames. Convergence (time vs. rmsd and time vs. Rg) was calculated using the *rms* and *gyrate* commands of GROMACS. Atomic fluctuations were calculated using the *rmsf* command of GROMACS. Principal component analysis was independently performed on the C-alpha atoms of the ED-OD (residues 11–157) and OD-AD (residues 119–285) using the *covar* and *anaeig* commands of GROMACS. The cosine contents for the analyzed trajectories were calculated to demonstrate PCA convergence with the *analyze* command of GROMACS. Porcupine plots were generated using the extremes identified for PC1–PC5 by the *anaeig* command of GROMACS. An in-house script was written to draw a vector from C-alpha atoms of the first extreme to equivalent C-alpha atoms in the last extreme in the bild format for visualization with UCSF Chimera [48]. Frames (coordinates) pertaining to ssDNA and ADP were extracted using the *trjconv* command of GROMACS. The rmsd calculations for the four ADPs were performed using UCSF Chimera [48]. The C-alpha of equivalent AD (residues 165–285) were aligned with the *match* function, and the ADP rmsd were calculated using the *rmsd* command of UCSF Chimera. The *rmsd* command evaluates the rmsd without any fitting. Similarly, the C-alpha atoms of the OD hexamer (residues 119–157) from each set of coordinates were aligned onto C-alpha atoms of the OD hexamer average (generated by the *anaeig* command of GROMACS during PCA) using the *match* command of UCSF Chimera. The *rmsd* between equivalent AD (C-alpha atoms) was then calculated using the *rmsd* command of UCSF Chimera. Clustering for the ED-OD and OD-AD was performed using the Quality Threshold algorithm with a threshold of 1.8 Å. The *rmsd* between the centroid representing the largest cluster and the remaining frames was calculated to determine the structural diversity within the analyzed trajectories. Measuring the AD staircase arrangement was performed using UCSF Chimera. The C-alpha atoms of the OD hexamer for each trajectory were aligned onto the symmetrized Rep using the *match* command of UCSF Chimera. A plane was drawn through the center of the six Lys240 for each coordinate and the symmetrized structure using the *define* command of UCSF Chimera. The angle between these two planes was calculated using the *angle* command of UCSF Chimera.

### 2.4. Figure Synthesis

Ribbon and licorice models of Rep were generated using UCSF Chimera [48]. Box scatter (histograms), line scatter, line, and bar plots were generated using Microcal Origin 2015.

## 3. Results

### 3.1. Each All-Atom MD Simulation of the PCV2 Rep Achieves Convergence within 200 nsec

**Figure 1:**
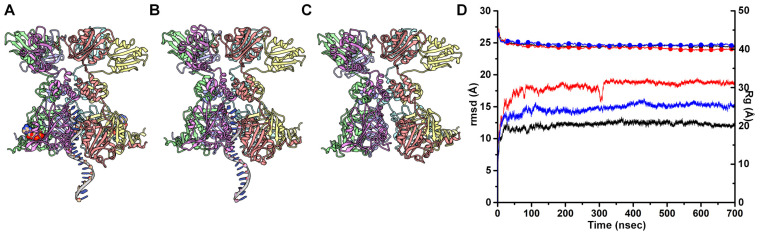
Molecular Dynamics simulation of PCV2 Rep. (**A**) Ribbon cartoon of full-length PCV2 Rep bound to ADP (CPK model) and 20 nt ssDNA. (**B**) Rep bound to 20 nt ssDNA and (**C**) Rep. Chains are colored purple (chain A at top), green (chain B), magenta (chain C), cyan (chain D), yellow (chain E), and red (chain F). (**D**) Time vs. rmsd in bold lines (Rep-(ssDNA + ADP) in black, Rep-ssDNA in red, and Rep in blue); time vs. Rg (radius of gyration) in thin lines and circles (same color as time vs. rmsd).

We performed a 700 nsec simulation for each of the following systems: (1) PCV2 Rep bound to a 20 nt ssDNA and four molecules of ADP + Mg^2+^, (2) PCV2 Rep bound to 20 nt ssDNA, and (3) PCV2 Rep (Figure 1A–C). We checked for convergence of simulation by plotting time versus root mean square-deviation (rmsd) and time versus radius of gyration (Rg) (Figure 1D). The plot shows that both the rmsd and Rg stabilize within the first 200 nsec of simulation. This suggests that the three systems have converged. However, the difference in rmsd and Rg between the three simulations suggests that the three systems may have adopted different conformations. We asked the following question: are the differences in rmsd and Rg due to the ED, OD, and AD adopting distinct arrangements with respect to one another (i.e., global flexibility)? To determine if the difference in conformations is due to the domains, we performed the same analyses for the hexamer of each domain separately (Figure S1). ED hexamers differ most when comparing the three systems. Their rmsd plateau is 15 Å for Rep-(ssDNA + ADP), 23 Å for Rep-ssDNA, and 20 Å for Rep. This difference supports the notion that ED for the three systems likely adopts different arrangements. However, their Rg values are comparable (~40 Å). The OD hexamer rmsd and Rg values for the three systems approach comparable values (3 Å for rmsd and 20 Å for Rg). The AD hexamers reach slightly different rmsd (4 Å, 5 Å and 5 Å) and Rg (34 Å, 33 Å, and 34 Å) values. The large rmsd values observed in Figure 1D are due to the ED adopting different positions than the starting model (Figure S1). The starting model was generated using a bacterial homologue of Rep since the ED was too mobile to observe with cryo-EM [18]. Indeed, when separately comparing the ED, OD, and AD, both the OD and AD exhibit less than 5 Å rmsd compared to the starting model (Figure S1). The differences between the Rg values are too small to draw any conclusion, and closer inspection of the trajectories are needed (see below). Overall, the rmsd and Rg plots indicate that the simulations have converged, and that Rep appears to be exhibiting global flexibility.

### 3.2. Flexible Regions of Rep Are Known to Be Functionally Important

**Figure 2:**
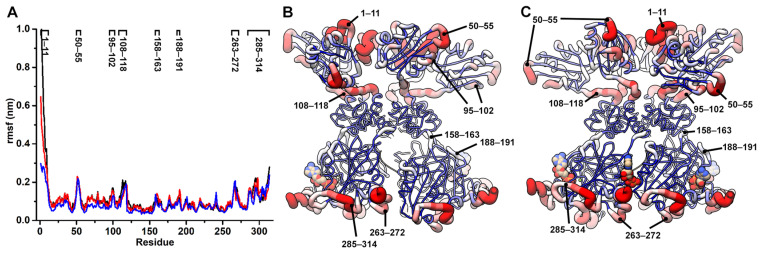
Root mean square fluctuations (rmsf) of PCV2 Rep. (**A**) Residue vs. rmsf of Rep-(ssDNA + ADP), Rep-ssDNA, and Rep using same colors as Figure 1. The traces are averages of the six chains of Rep calculated from the last 200 nsec of each simulation. Local regions with high rmsf are labeled on top. (**B**) Licorice model of Rep color coded according to the rmsf values, with high rmsf in red, medium in white, and low in blue. The thickness of licorice is also drawn according to the rmsf values. (**C**) Ninety-degree rotation of Rep about the y-axis.

To determine if and how the binding of biomolecules (ssDNA and ADP) affect the dynamics of Rep, we performed root mean square fluctuation (rmsf) analysis for each simulation. It occurred to us that the potential movement of domains, with respect to one another, may create noise within the rmsf profile if the entirety of the polypeptide chains were to be analyzed. This is because the rmsf alignment algorithm optimizes the alignment of bodies by distributing diversity throughout the entirety of the bodies. Consequently, to decouple the potential movement of each domain from the local motion within each domain, we performed rmsf analyses for each domain of each chain independently. The rmsf profiles for all six chains within each simulation are nearly indistinguishable (Figure S2) and can thus be averaged (Figure 2). The three simulations exhibit comparable rmsf profiles; thus, the binding of ssDNA and ADP to Rep does not appear to affect the local flexibility of Rep in this set of experiments. The three rmsf profiles consistently demonstrate the following regions to exhibit higher fluctuations with respect to the baselines: (1) residues 1–11 (N-terminus), (2) residues 50–55 (binds nona-nucleotide at *ori*), (3) residues 95–102 (binds nona-nucleotide at *ori*), (4) residues 108–118 (ED-OD linker), (5) residues 158–163 (the OD-AD linker), (6) 188–191 (interacts with the OD-AD linker), (7) residues 263–272 (loop near nucleotide binding site Arg-finger, Arg276/277), and (8) residues 285–314 (C-terminus). It is interesting to note that the N-termini of Rep simulation demonstrates less fluctuations than when Rep is bound to ssDNA and ssDNA + ADP.

### 3.3. The ADP Nucleotide in the DE Interface Is Ready for Release

**Figure 3:**
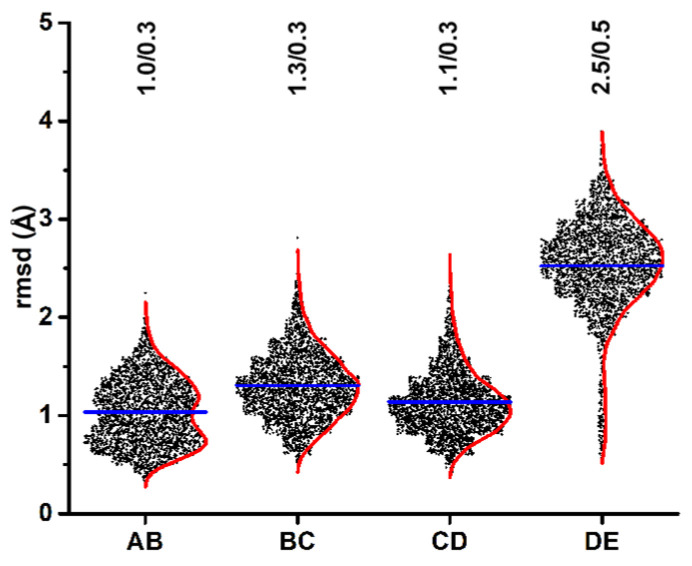
rmsd-histograms (box scatter plots) of the four nucleotide binding sites of Rep at the AB, BC, CD, and DE interfaces. The average and kernel smooth for each histogram are shown as blue and red lines, respectively. The average and standard deviation values for each histogram is written at the top. The AB interface exhibits a bimodal distribution, although the mean of the two peaks are less than 1 Å apart.

Our cryo-EM structure demonstrated that the buried surface area, number of hydrogen bonds, and salt bridges between ADP and Rep was strongest at the AB interface and progressively weaker as one descends down the staircase -with the DE interface having the least number of interactions with ADP and thus proposed to release ADP in the next cycle of ATP binding, hydrolysis, release, and ssDNA translocation. To determine if this structure-based proposition agreed with our MD results, we measured the rmsd of ADP at interfaces AB, BC, CD, and DE for the last 200 nsec of simulation with respect to the 500 nsec frame. The rmsd histograms clearly demonstrate that ADP displacement is comparable for the AB, BC, and CD interfaces; however, ADP in the DE interface has significantly higher rmsd values (Figure 3). The greater rmsd values at the DE interface support our proposition that the DE interface is likely to release the ADP in the next cycle of ATP binding and hydrolysis [18]. Similar results were obtained when the alternative ADP binding subunit (e.g., AD of chain B of the AB interface) was overlaid onto the corresponding domain of the 500 nsec frame. The bimodal distribution observed at the AB interface may be insignificant given the small difference in length between the two peaks; however, further studies are needed to explore this observation.

### 3.4. Essential Dynamics of Rep Demonstrates Global Flexibility

The time vs. rmsd analyses suggested that the ED and AD of Rep were mobile (Figure 1 and Figure S1). To pursue this possibility in greater detail, we used Principal Component Analysis (PCA) to reduce the high dimensionality present in the three simulations and describe the essential dynamics of Rep. We measured the cosine content of each trajectory to demonstrate that convergence had been reached (Figure S3) [49]. To simplify interpretation of the PCA, we performed the analysis on the ED-OD and OD-AD sections separately. We also truncated the N-(residues 1–11) and C-termini (residues 285–314) from the analyses due to their high mobility (Figure 2). For the ED-OD section, scree plots describing the contribution of each PC to the motions indicate that the first three components describe less than 65% of the motion. Elbows can be observed after the PC4 of Rep-(ssDNA + ADP) simulation and after the PC5 of the Rep-ssDNA and Rep simulations. Indeed, five components are required to describe up to 78% of the data for the three simulations (Figure S4A–C). Scree plots for the OD-AD section indicate that the first three principal components describe up to 60% of the motion. Elbows can be observed after the PC5 of Rep-(ssDNA + ADP) simulation, after the PC4 of the Rep-ssDNA simulation, and after the PC3 of Rep simulations. Indeed, five components are required to describe up to 73% of the motion for the three simulations (Figure S3D–F).

**Figure 4:**
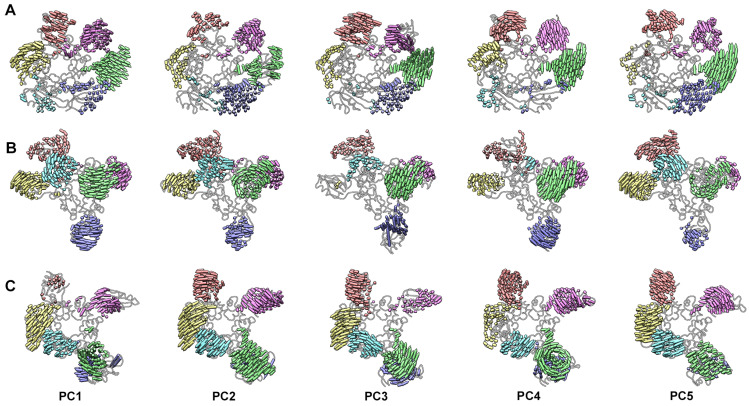
Porcupine plots from PCA of the ED-OD from the three simulations. The pegs (vectors) are color coded according to Figure 1. Only pegs longer than 2 Å are shown. PCV2 is shown as a licorice with silhouettes. ED-OD is shown with ED closest to and OD distal to the viewer. Shown are PC1-PC5. (**A**) Rep-(ssDNA + ADP), (**B**) Rep-ssDNA, and (**C**) Rep.

**Figure 5:**
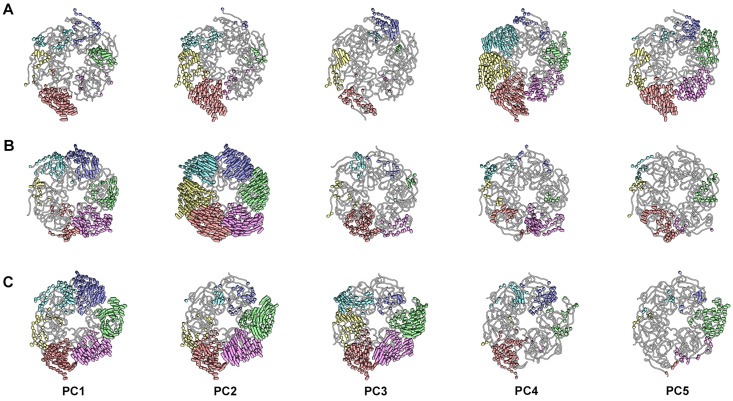
Porcupine plots from PCA of the OD-AD from the three simulations. The pegs (vectors) are color coded according to Figure 1. Only pegs longer than 2 Å are shown. PCV2 is shown as a licorice with silhouettes. OD-AD is shown with AD closest to and OD distal to the viewer. Shown are PC1-PC5. (**A**) Rep-(ssDNA + ADP), (**B**) Rep-ssDNA, and (**C**) Rep.

We describe the essential dynamics of Rep using porcupine plots created from PC1–PC5 (Figure 4, Figure 5, Figures S5 and S6). Porcupine plots use vectors (shown as pointy pegs) to communicate the movement of C-alpha atoms from one extreme to the other for each PC. In all cases, ED demonstrates large motion, and OD demonstrates motion less than 2 Å (Figure 2 and Figure 4). We describe the ED motions with respect to the viewer and a plane that runs through the six OD, referred to as the OD plane. Rep is oriented with the ED closest and OD furthest from the viewer. The movement of ED can be described as either toward the viewer, away from the viewer, or in the OD plane. A comparison of PC1–PC5 for all three simulations demonstrates a sophisticated range of movements (Figure 4). For the Rep-(ssDNA + ADP) simulation, PC1 shows ED^A,C,D,E,F^ (ED of chains A, C, D, E, and F) to rotate and shift away from the viewer, while ED^B^ rotates and shifts toward the viewer (compare top to side views) (Figure 4 and Figure S5). PC2 shows ED^A,B,C,D^ to move away from the viewer, and ED^E^ and ED^F^ rotate clockwise (*C*) and counter-clockwise (*CC*) in the OD plane, respectively. PC3 shows ED^A,B^ and ED^C,F^ to rotate toward and away, respectively, from the viewer, and ED^D,E^ to rotate *C* in the OD plane. PC4 shows ED^A^ and ED^B,C,D,E^ to rotate toward and way from the viewer, respectively. ED^F^ rotates *C* in the OD plane. PC5 shows ED^A^ and ED^C,F^ to rotate away from and toward the viewer, respectively. ED^B,D,E^ rotates *CC* in the OD plane. It can be generalized from PC1–PC5 that ED^A,B,F^ exhibit the greatest mobility, while ED^C,D^ are the least mobile. For the Rep-ssDNA simulation, PC1 shows ED^A,B,C,E^ and ED^F^ to rotate away from and toward the viewer, respectively. ED^D^ rotates *CC* in the OD plane. PC2 shows ED^A,D,F^ and ED^C^ to rotate away from and toward the viewer, respectively. ED^B,E^ rotates *C* in the OD plane. PC3 shows ED^A,C^ and ED^D^ to rotate toward and away from the viewer, respectively. ED^B,F^ rotates *C* in the OD plane. ED^E^ moves very little. PC4 shows ED^A,F^ and ED^B,C^ to rotate away from and toward the viewer, respectively. ED^E^ rotates C in the OD plane. ED^D^ moves very little. PC5 shows ED^A,B^ to rotate *CC* in the plane of OD. ED^C,F^ and ED^D,E^ rotate toward the viewer, respectively. It can be generalized that ED^A,C^ are the most mobile, and ED^D^ is the least mobile. For the Rep simulation, PC1 shows ED^A,D,F^ and ED^B,C^ to rotate toward and away from the viewer, respectively. ED^E^ rotates *C* in the plane of OD. PC2 shows ED^A,D,E^ to rotate toward the viewer and ED^B,C,F^ to rotate *C* in the plane of OD. PC3 shows ED^A,C^ and ED^D,E,F^ rotate toward and away from the viewer, respectively. ED^B^ rotates *CC* in the plane of OD. PC4 shows that ED^A,C^ rotates toward the viewer. ED^B,F^ and ED^D,E^ rotate *C* and *CC* in the OD plane, respectively. PC5 shows that ED^A^ and ED^B,F^ rotate *CC* and *C* in the OD plane, and ED^D,E^ rotates away from the viewer. It can be generalized that ED^A,C^ are the most mobile, while ED^F^ is the least mobile. A comparison of ED positions and dynamics in the three simulations clearly demonstrates global flexibility. This agrees with the cryo-EM studies that were unable to identify the location of ED in the averaged structure [18].

PCA analyses for the OD-AD section shows AD to be less mobile than ED (Figure 5 and Figure S6). We visualize the essential dynamics by orienting Rep such that AD is closest and OD is furthest from the viewer. We describe the motions of AD with respect to the viewer, the OD plane, and an axis that is normal to the OD plane -referred to as the OD axis. Comparison of PC1–PC5 demonstrates a diverse range of movements where either one, multiple, or all AD move more than 2 Å. For the Rep-(ssDNA + ADP) simulation, PC1 shows AD^A,B,C,D,E,F^ to rotate *C* in the OD plane. PC2 shows AD^A,B,C^ to move very little, AD^D,E^ to move away from and AD^F^ to move toward the OD axis. PC3 shows AD^A,B,D^ to move very little, AB^E,F^ to rotate toward each other and away from the viewer, and AD^C^ to rotate *C* in the OD plane. PC4 shows AD^A,E,F^ to move toward the OD axis, AD^B,C^ to move away from both the OD axis and the viewer, and AD^D^ to move toward the OD axis. PC5 shows AD^A,F^ and AD^B,C^ to move toward and away from the OD axis, respectively, and AD^D,E^ rotates *C* in the OD plane. For the Rep-ssDNA simulation, PC1 shows AD^A,C,D^ to move toward the OD axis, AD^B^ rotates *C* in the OD plane, and AD^E,F^ moves very little. PC2 shows AD^A,B^ to rotate *C* around the OD axis and toward the viewer, AD^C^ and AD^D^ move toward and away from the OD axis, respectively, and AD^E,F^ rotates *C* in the OD plane. PC3 shows AD^A,D^ and AD^F^ to move toward and away from the OD axis, respectively, and AD^B,C,E^ moves little. PC4 shows AD^A^ to rotate toward and AD^D,F^ to rotate away from the OD axis, respectively, and AD^B,C,E^ to move little. PC5 shows AD^A,C,D,E^ to move little, AD^B^ to move away from the OD axis, and AD^F^ to rotate *CC* in OD plane. For the Rep simulation, PC1 shows AD^A^ to move away from and AD^C^ to move toward the OD axis, respectively, and AD^B^ and AD^D,E^ move *C* and *CC* in OD plane. AD^F^ moves away from the viewer. PC2 shows AD^A^ to move away from the OD axis and toward the viewer. AD^B^ and AD^E,F^ move *CC* in the OD plane, and AD^C,D^ moves little. PC3 shows AD^A^ to move *CC* and toward the viewer, AD^B,E^ and AD^D^ to move *C* and *CC* in the OD plane, respectively, and AD^F^ to move away from the OD axis and toward the viewer. PC4 shows AD^A,B,E^ to move little, AD^C^ and AD^D^ to rotate *C* and *CC* in the OD plane, respectively, and AD^F^ to move toward the OD axis and the viewer. PC5 shows AD^A,C,D,E,F^ to move little and AD^B^ to move toward the OD axis.

We then measured the extent of rotation and translation needed to overlay domains from the extremes identified by PCA. These measurements provide the extent to which the ED and AD move during the last 200 nsec of simulation. For the Rep-(ssDNA + ADP) simulation, the ED rotates up to 49° (chain B, PC2) and translates nearly 6 Å (chain B, PC5). The AD rotates 9° (Chain F, PC1) and translates ~3 Å (ChainE, PC4). For the Rep-ssDNA simulation, the ED rotates as much as 28° (Chain C, PC3) and translates close to 3 Å (Chain B, PC4), and the AD rotates as much as 15° (Chain D, PC2) and translates less than 3 Å (Chain F, PC2). For the Rep simulation, the ED rotates up to 37° (Chain C, PC3) and translates more than 7 Å (Chain B, PC1), and the AD rotates as much as 14° (Chain B, PC2) and translates less than 1.5 Å (Chain F, PC2 and PC3).

### 3.5. ADP and ssDNA Alter the Global Flexibility of AD

**Figure 6:**
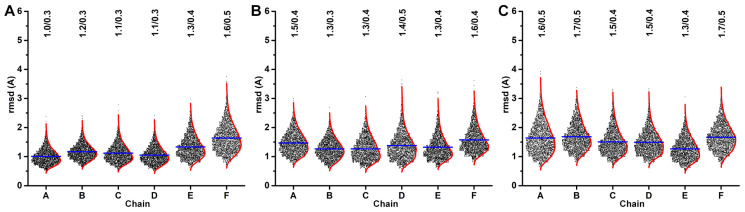
rmsd histograms (box scatter plots) of each AD chain after superposition of the OD hexamer. The average and kernel smooth for each histogram are shown as a blue and red lines, respectively. The average and standard deviation values for each histogram is written at the top. (**A**) Rep-(ssDNA + ADP), (**B**) Rep-ssDNA, and (**C**) Rep simulations.

The binding of ADP and ssDNA do not appear to affect the local flexibility of Rep. Therefore, we asked the following question: do these biomolecules affect the global flexibility of Rep? To answer this question, we measured the displacement (rmsd) of each AD with respect to an OD-AD average. We extracted the Rep coordinates for each trajectory frame, aligned the OD hexamer of the extracted coordinates to the corresponding OD hexamer of the PCA generated average, and measured the rmsd between the extracted AD and the corresponding AD of the PCA generated average (Figure 6A–C). The histograms demonstrate deviations of the AD from the average coordinate identified by PCA. Consequently, the average of rmsd for each AD is comparable to the rmsf value for that AD. For the Rep-(ssDNA + ADP) simulation, the rmsd histograms for AD^A,B,C,D^ exhibit comparable averages and averages that are smaller than the averages for AD^E,F^. The larger averages for AD^E,F^ indicate that these domains exhibit greater global flexibility. The AD at the bottom of the AD staircase (AD^F^) exhibits the greatest global flexibility. For the Rep-ssDNA simulation, the rmsd histograms for the AD^B,C,D,E^ are comparable, whereas the rmsd histograms for the AD^A,F^ are comparable. The larger averages for AD^A,F^ suggest that these domains exhibit greater global flexibility. For the Rep simulation, the rmsd histograms for AD^A,B,C,D,F^ are comparable to one another, suggesting that these domains exhibit comparable global flexibility. The smaller average for AD^E^ suggests that this domain is closer to the PCA average. Overall, the average rmsd values for each AD increase when the ADP nucleotide is removed, and then again when the ssDNA is removed.

**Figure 7:**
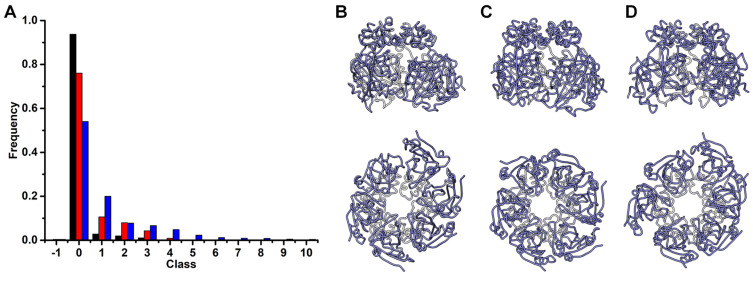
Quality Threshold clustering. (**A**) Bar graph showing the number of members per cluster at a quality threshold of 1.8 Å. The bars are color coded similar to Figure 1 (black: Rep-(ssDNA + ADP), red: Rep-ssDNA, blue: Rep). Licorice representations of centroids from first cluster of (**B**) Rep-(ssDNA + ADP), (**C**) Rep-ssDNA, and (**D**) Rep. Top panel are side views, and bottom panel are bottom views, the same orientation as Figure 6. The staircase arrangement is maintained in the largest cluster of the Rep-(ssDNA + ADP) and Rep-ssDNA but not Rep simulation.

To further explore the effect of ssDNA and ADP on the conformational states of Rep, we used Quality Threshold (QT) clustering on the OD-AD hexamer [50]. This method generates clusters with members that share rmsd values no greater than a user-defined threshold. However, low threshold values generate a large orphan cluster whose members share rmsd values greater than the user-defined threshold. Consequently, it is important to identify the proper threshold that diminishes the number of members in the orphan cluster. We searched for a single threshold that minimizes the number of orphaned conformations in the three simulations (Figure S7). We then compared the number of clusters and the number of members within each cluster generated by this threshold (Figure 7A). The number of clusters in the Rep-(ssDNA + ADP), Rep-ssDNA, and Rep simulations for a threshold of 1.8 Å are 4, 5, and 11, respectively. The largest class in each simulation accounts for 94%, 75%, and 54% of the frames (Figure 7B–D). A comparison of the centroid from the largest cluster to the remaining trajectory frames indicates that convergence has been reached (Figure S8). The QT clustering demonstrates that the number of classes increases and the number of members within each class decreases following the removal of ADP and then ssDNA. This supports the notion that the binding of ssDNA and ADP limits the conformational space sampled by Rep.

### 3.6. The ssDNA Is Responsible for the AD Staircase Arrangement

**Figure 8:**
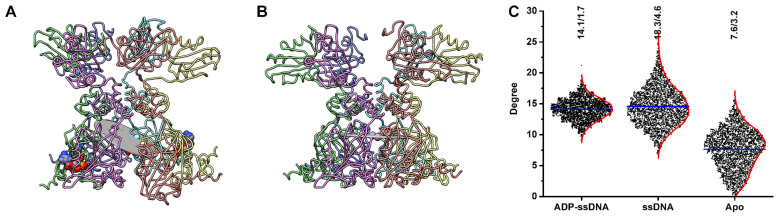
Staircase arrangement of the Rep AD. (**A**) Licorice model of the final frame from the Rep-(ssDNA + ADP) simulation. Rep is shown as licorice with silhouettes and color coded according to Figure 1. A plane is drawn through the centroids of Lys240 from each of the six chains and shown as a gray disk. (**B**) Symmetrized model of Rep with the plane through the six Lys240, shown as a gray disk. (**C**) Degree histograms (box scatter plots) representing the angles between the plane calculated for each simulation and a symmetrized Rep. The average and kernel smooth for each histogram are shown as a blue and red lines, respectively. The average and standard deviation values for each histogram are written at the top.

The cryo-EM structure of Rep bound to ssDNA and ADP visualize the AD to form a staircase around the ssDNA, as three residues (W202, Lys240, and Gly241) from each AD interact with the backbone phosphates of the ssDNA [18]. We asked the following question: are the factors responsible for the staircase arrangement a result of the bound ssDNA and ADP, ssDNA, and ADP, or the natural property of Rep? To answer this question, using our three MD simulations, we generated a plane that intersects the center of each Lys240 amino acid and measured the angle between this plane and a C_6_ symmetrized Rep (Figure 8A,B). For reference, the angle between the deposited cryo-EM structure (PDB entry 7LAR) and a computationally symmetrized (C_6_) Rep is 32°. The angular histograms of the Rep-(ssDNA + ADP) and Rep-ssDNA simulations have averages of 14° and 18°, whereas the average for the Rep simulation is 8°. This suggests that the AD staircase in the Rep-(ssDNA + ADP) and Rep-ssDNA simulations are maintained but lost in the Rep simulation. Indeed, the first cluster from the QT analysis supports this (Figure 7B–D).

## 4. Discussion

CRESS-DNA viruses are predicted to possess Reps with comparable structures [14,21]. Our cryo-EM structure of the PCV2 Rep was the first structure for this group of viruses [18]. Our studies demonstrated that Reps possess three structural domains: an ED that is highly mobile, an unexpected OD that is responsible for oligomerizing six chains of Rep, and an AD that belongs to the AAA+ SF3. The structure visualized the six ADs to form a staircase arrangement around the ssDNA bound to the central axis of the homo-hexamer and identified ADP bound between pairs of AD starting at the top of the staircase. Our biochemical studies demonstrated that Rep is capable of basal NTPase activity in the presence of ATP, GTP, CTP, and UTP. Using these observations, we proposed a hand-over-hand mechanism for ssDNA translocation by Rep, where sequential ATP binding, hydrolysis, and ADP + Pi release results in translocation of the AD at the bottom of the staircase to the top of the staircase, while the remaining five ADs translocated one step down as they pulled the ssDNA through the center of Rep [21]. We compared this model to the concurrent (also referred to as the all-or-none or inch worm) model, where all ADs concurrently bind, hydrolyze ATP, and release ADP + Pi to translocate their substrate through their central pore. To attain a better understanding of how Rep carries out ssDNA translocation, we performed a 700 nsec all-atom Molecular Dynamics simulation of a full-length Rep bound to ssDNA and ADP, Rep bound to ssDNA, and Rep. Our simulations converged within the 200 nsec of simulation; however, we decided to use the last 200 nsec of simulation for our analyses. Our simulations identified local flexible regions that are known to be important in the function of Rep. These include loops (50–55, and 95–102) that are responsible for recognizing and processing PCV2’s origin of replication and a loop near the nucleotide binding site Arg-fingers (Arg276 and Arg277) that may be responsible for sensing the nucleotide state. The simulations also identified flexible local regions that require further investigation for their role in ssDNA translocation. These include the positively charged N-terminus (residues 1–11) that may bind the dsDNA at the replication fork, the ED-OD linker (108–118), whose flexibility is likely responsible for ED’s mobility and possible importance for ssDNA translocation, the OD-AD linker (158–163), whose flexibility allows the AD to translocate from the bottom to the top of the staircase, and the C-terminus (285–314), which may also be responsible for nucleotide recognition. Consequently, our MD simulations identify regions that may play pivotal roles in the translocation of ssDNA.

The essential dynamics of Rep shows that the ED and AD move in complex patterns. The first five PC indicate that the ED and AD can move in the plane of the OD, toward and away from the OD, or a combination of both. Rotations and translations were found as large as 49° and 7 Å for ED, and 15° and 3 Å for AD. ED’s ability to demonstrate a much larger range of motion may be due to the lack of an ED–ED interface. Indeed, biochemical and structural studies have demonstrated that an isolated PCV2 ED is a monomeric protein even at high concentrations [51]. The simulations also demonstrate that the binding of ssDNA and nucleotides to AD do not affect the local flexibility of Rep. Hence, it seems that the properties of ED may be independent of AD and its ligands; however, this needs further investigation. It also remains to be seen if the ED maintains this degree of conformational variability during dsDNA unwinding and ssDNA translocation. We note that while the OD ring shows little movement in our simulations, it remains to be seen if it experiences greater movement as ssDNA is translocated through its central pore. Overall, our inability to visualize the ED in our cryo-EM experiments, the extensive motion of the ED observed within each simulation, and the distinct arrangements of the ED when comparing the three simulations are consistent with one another. With regards to role of ssDNA and ADP in the movement and conformational variability of AD, the simulations demonstrate that the binding of ssDNA to Rep is responsible for the observed staircase arrangement of AD in the cryo-EM structure. The simulations also demonstrate that the binding of ADP to Rep-ssDNA restricts the mobility of AD, most likely because the nucleotide stabilizes the AD-AD interface. Consequently, while the binding of ssDNA and ADP do not affect the local flexibility of Rep, they do restrict the global flexibility of AD. It remains to be seen how the binding of ATP and ADP + Pi affects the dynamics of Rep. Consequently, our MD simulations identify regions that may play pivotal roles in the translocation of ssDNA.

## 5. Conclusions

We have used Molecular Dynamics simulation to study the conformations and dynamics of a CRESS-DNA Rep. Our studies demonstrate that while the binding of nucleotides does not appear to affect the local flexibility of Rep, they do affect the global flexibility of Rep (i.e., the binding of nucleotides and ssDNA restrict the conformational states accessed by Rep). Using PCA, we demonstrated that Rep undergoes sophisticated conformational changes that require five PC to be described. Our studies also demonstrate that it is the binding of ssDNA that is responsible for maintaining the staircase arrangement of the AD observed in our earlier reported cryo-EM structure [18]. We conclude by discussing that pentameric, hexameric, and octameric oligomers of proteins that utilize AAA+ motors for translocating biomolecules through their central pore have been described [40,52]. Moreover, the hand-over-hand versus the concurrent model are not exclusive to an oligomeric state of a ringed motor. For example, bacteriophage Phi29 uses gp16, a pentameric ATPase motor, to package dsDNA into the phage capsid. Using single-molecule optical tweezers, investigators recently proposed gp16 to use a concerted model where concurrent ATP binding followed by concurrent ATP hydrolysis results in the AD to alternate between a staircase and a planar arrangement [53]. This same model has also been reported for the translocation of polypeptide through the hexameric *Plasmodium* translocon of exported proteins (PPTEX) that transports *Plasmodium* proteins across the *Plasmodium* membrane into the host erythrocytes [54]. Thus, it remains to be described why different oligomeric states exist, how the same motor can be utilized in different oligomeric arrangements, and why two different models of translocation are used.

## Data Availability

Data are contained within the article and supplementary materials.

## Supplementary Materials

The following supporting information can be downloaded at: https://www.mdpi.com/article/10.3390/v15122393/s1, Figure S1: Domain rmsd vs. time and Rg vs. time; Figure S2: Chain rmsf-vs. residue; Figure S3: Cosine content analysis; Figure S4: Scree plots; Figure S5: PCA porcupine plots of ED-OD; Figure S6: PCA porcupine plots of OD-AD; Figure S7: QT threshold determination; Figure S8: Centroid rmsd vs. time.
