# Supplementary material for: Dynamics and Conformations of a Full-Length CRESS-DNA Replicase
Source: Viruses. 2023 Dec 8;15(12):2393. doi: 10.3390/v15122393 (PMC10747457; doi:10.3390/v15122393)
Supplement: Supplementary file 1 [file viruses-15-02393-s001.zip › viruses-2714728-supplementary.pdf]

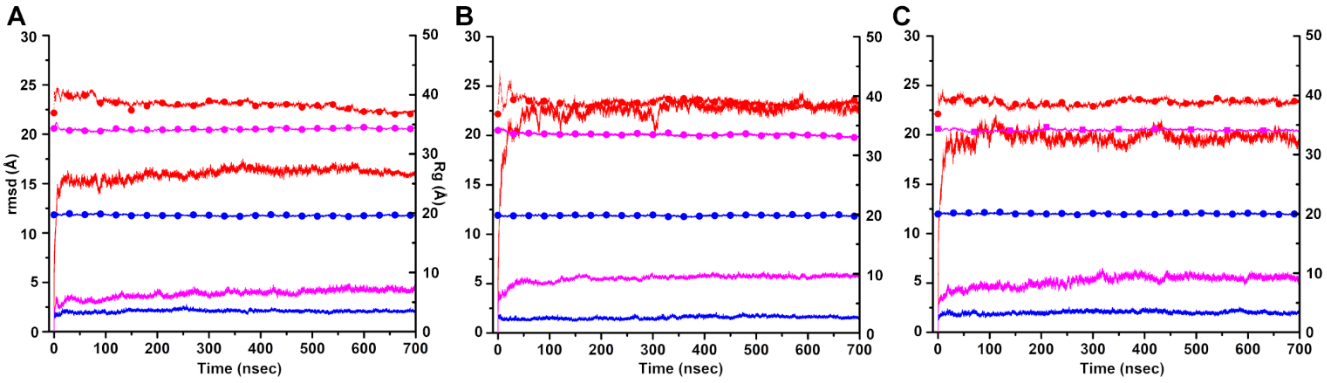

Figure S1. Domain rmsd vs time and Rg vs time. Global flexibility of Rep suggested by ED and AD hexamers from the three simulations reach different rmsd values. Time vs rmsd are shown as line plots, and time vs Rg are shown as line+scatter plots. ED are shown in red, OD in blue, and AD in magenta. A) Rep-(ssDNA+ADP), B) Rep-ssDNA, and C) Rep.

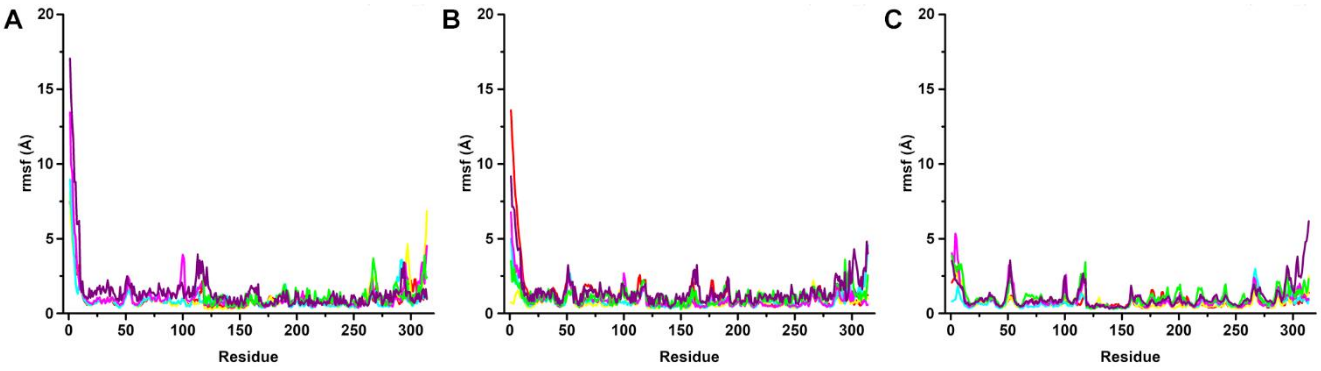

Figure S2. Chain rmsf vs residue. Profiles are comparable within each simulation. The traces are colored according to the subunits in Figure 1. A) Rep-(ssDNA+ADP), B) Re-ssDNA, C) Rep.

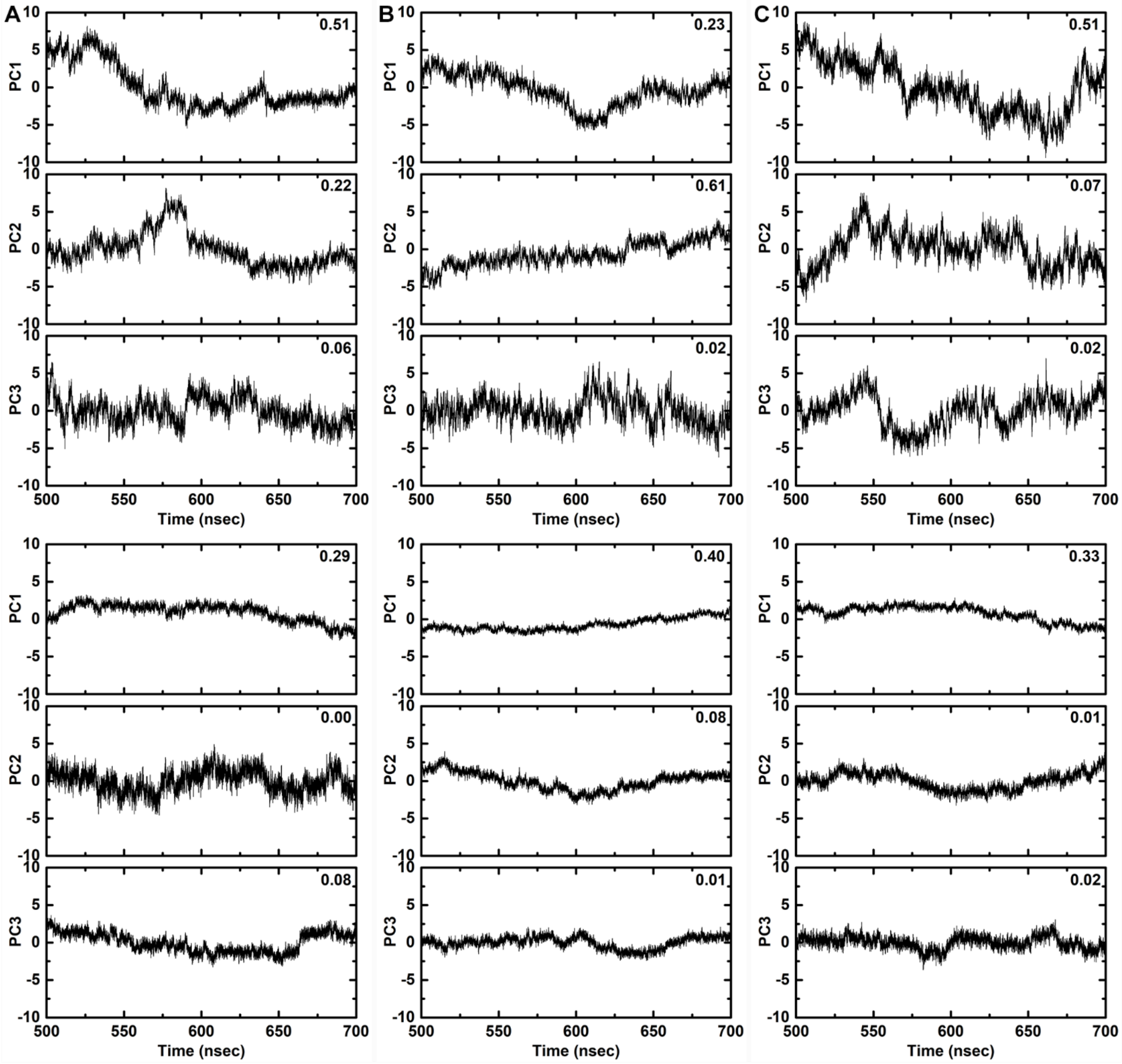

Figure S3. Cosine content analysis. The first three PC for the 500 to 700 nsec of simulation analyzed in this study. The first three PC of the A) Rep-(ssDNA+ADP), B) Rep-ssDNA, and C) Rep trajectories. The cosine content values are shown on the top right of each panel.

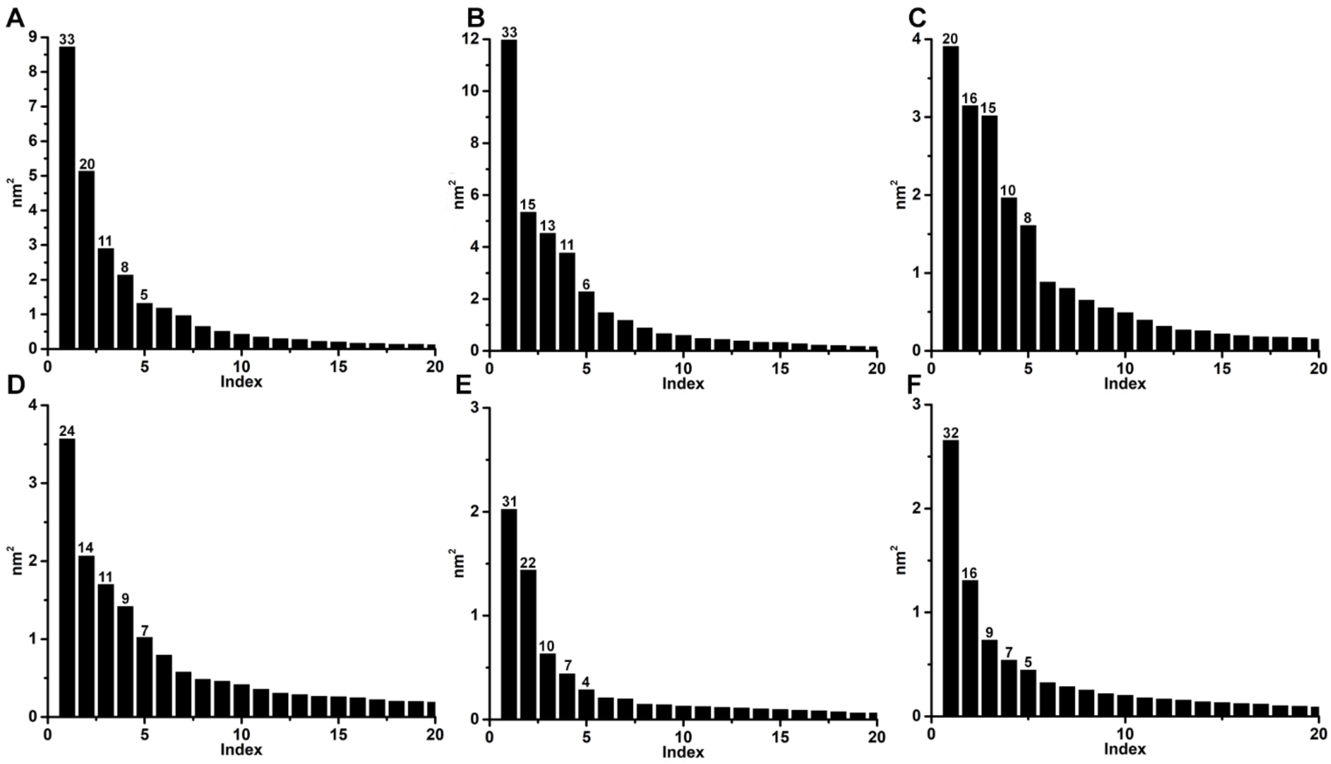

Figure S4. Scree plots. Derived from principal component analysis (PCA) of Rep. Numbers on top are the summative variance. A) PCA of Rep-(ssDNA+ADP) ED-OD, B) PCA of Rep-ssDNA ED-OD, C) PCA of Rep ED-OD, D) PCA of Rep-(ssDNA+ADP) OD-AD, E) PCA of Rep-(ssDNA) OD-AD, and F) PCA of Rep OD-AD.

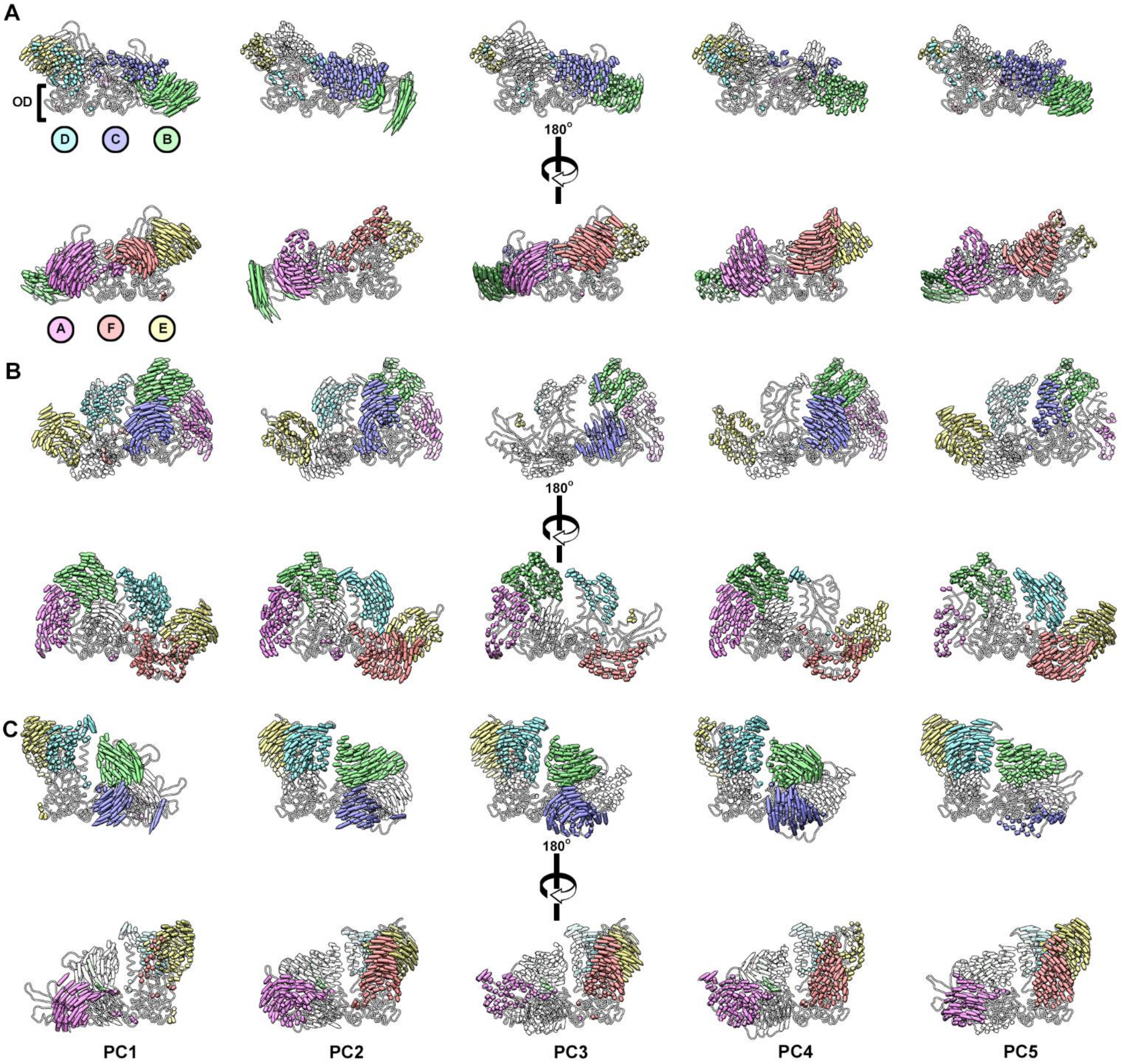

Figure S5. PCA porcupine plots of ED-OD. The schematics are the same as Figure 5. Showing side views of the ED-OD section. The subunits are labeled at the bottom of the figure for clarity. The top half shows subunits BCD, and bottom half shows subunit EFA. A) Rep-(ssDNA+ADP), B) Rep-ssDNA, C) Rep.

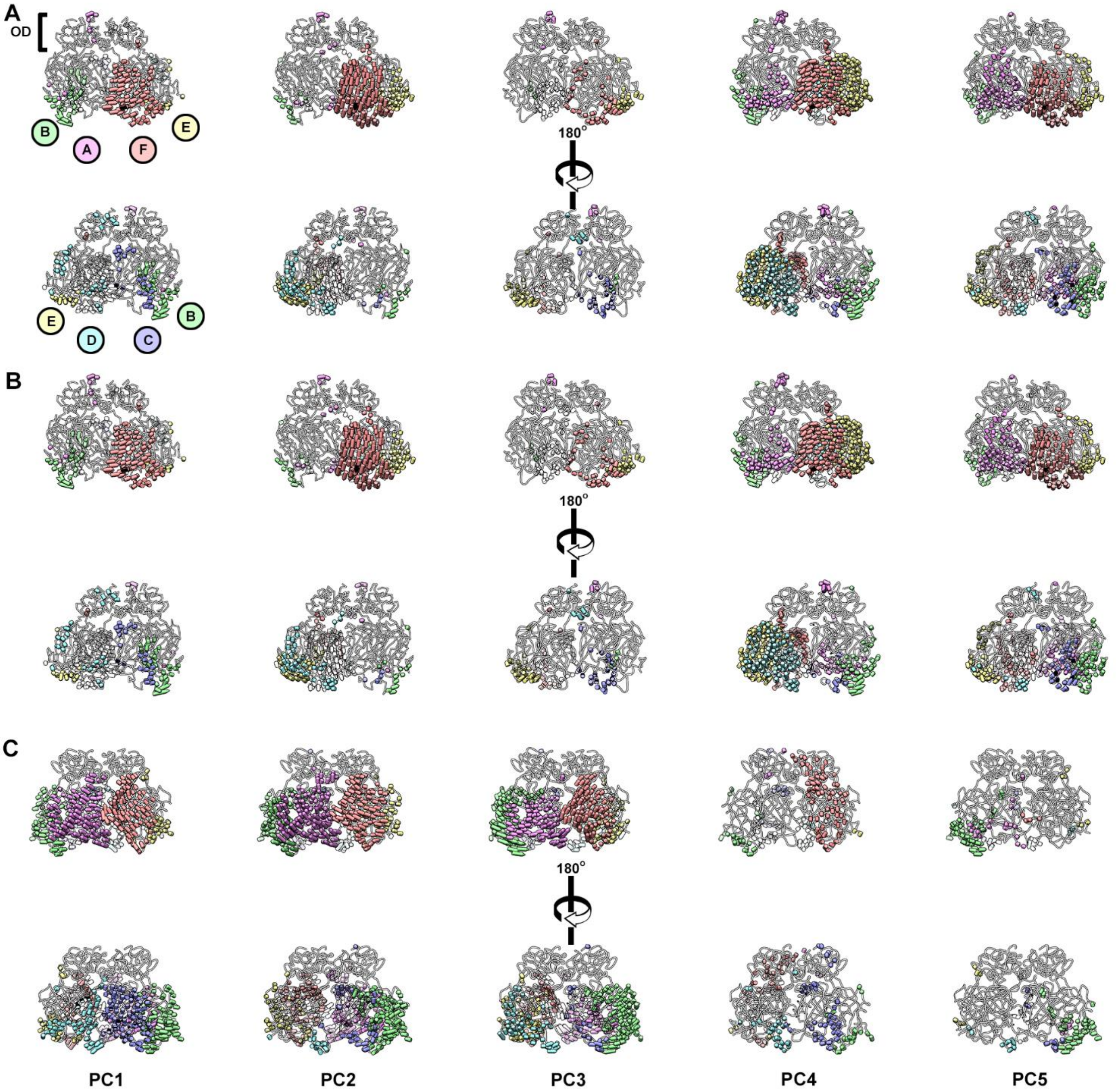

Figure S6. PCA porcupine plots of OD-AD. The schematics are the same as Figure 6. Showing side views of the OD-AD section. The subunits are labeled at the bottom of the figure for clarity. The top half shows subunits ABEF, and bottom half shows subunit BCDE. A) Rep-(ssDNA+ADP), B) Rep-ssDNA, C) Rep.

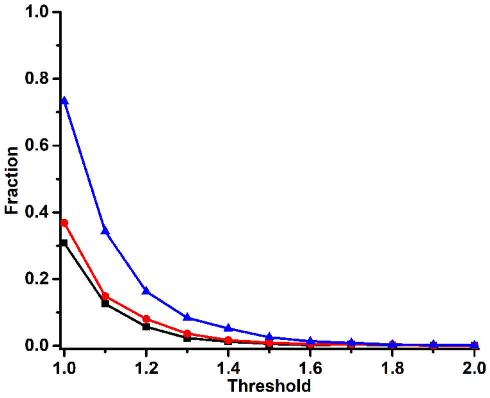

Figure S7. QT threshold determination. Threshold values and the resulting percentage of frames that are assigned to the orphan cluster for each simulation. Rep-(ssDNA+ADP) in black, Rep-ssDNA in red, and Rep in blue.

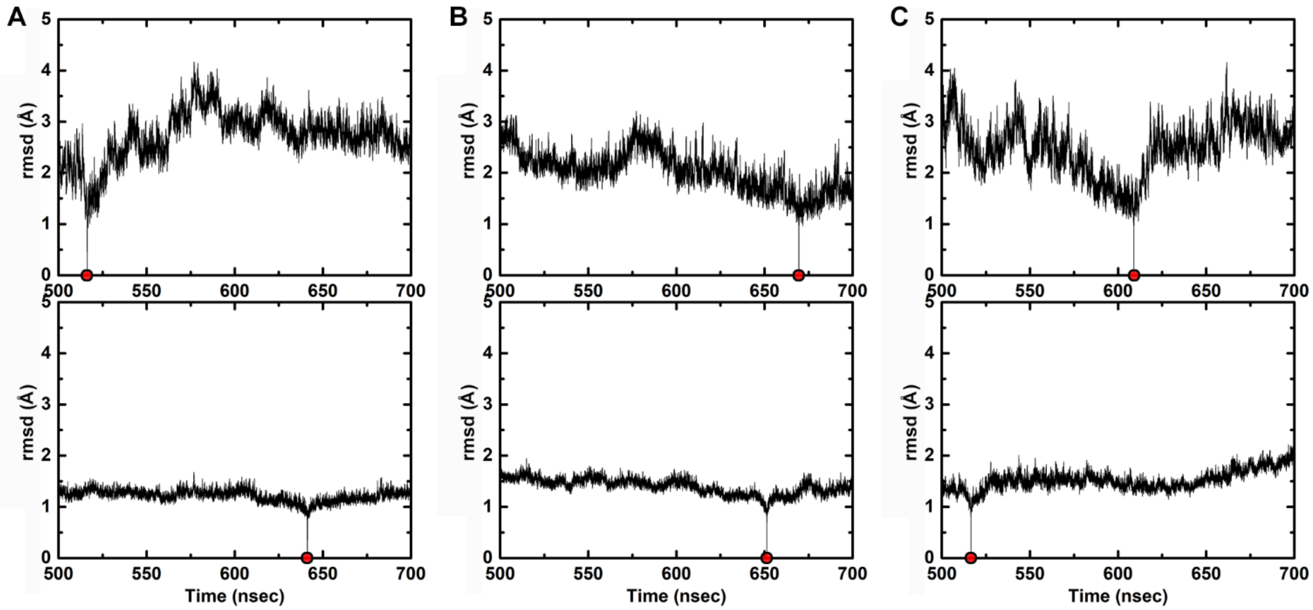

Figure S8. Centroid rmsd vs time. Plots compare the coordinates of the first QT cluster centroid (red circle) to frames 500 to 700 nsec of the simulation. A) Rep-(ssDNA+ADP), B) Rep-ssDNA, C) Rep. The top panel represents the ED hexamer, and bottom panel represents the AD hexamer.
